# Supplementary material for: Content-rich biological network constructed by mining PubMed abstracts
Source: BMC Bioinformatics. 2004 Oct 8;5:147. doi: 10.1186/1471-2105-5-147 (PMC528731; doi:10.1186/1471-2105-5-147)
Supplement: Additional File 5 — The original Chilibot query results of the term "long-term potentiation (LTP)" and 22 other terms, limiting the latest references analyzed to the years 1990, 1995, 2000, and 2004. [file 1471-2105-5-147-S5.bz2 › chilibotAdditionalFile5/ltp2004/html/TRKB_AMPA.html]

 


 **TRKB** and **AMPA** 
  
Found 14 abstracts in PubMed,  **14 abstracts were retrieved and analyzed**.  


---

 Search Google  |
 PDF files only 
|  EDU domain only 

---

**Interactive relationship** (e.g. stimulation, inhibition, etc)

- Moreover, analysis of  **TrkB**  expression in culture revealed a close correlation between the percentage of nTS neurons in which BDNF inhibits  **AMPA**  currents and the percentage of neurons that exhibit  **TrkB**  immunoreactivity.  Ref: 10684891 J Neurosci, 2000
- Thus, the impaired  **AMPA**  receptor function in the stg mutant GC is not likely to result from the reduced BDNF  **TrkB**  signaling.  Ref: 10407040 J Neurosci, 1999
- Our results demonstrate that biarylpropylsulfonamide  **AMPA**  receptor potentiators are capable of modulating the expression of BDNF and  **TrkB**  mRNA in a dose and time dependent manner.  Ref: 12213254 Neuropharmacology, 2002

**Parallel relationship** (e.g. studied together, co-existance, homology, etc.)

- In the present study, we investigated the effect of a novel  **AMPA**  receptor potentiator LY404187 and its active isomer LY451646 on the expression of BDNF protein and mRNA, as well as  **TrkB**  mRNA in rat hippocampus.  Ref: 12213254 Neuropharmacology, 2002
- To explore the translocation mechanism of individual  **AMPA**  receptor subunits, we utilized the human embryonic kidney 293 cells carrying the BDNF receptor  **TrkB** .  Ref: 12130635 J Biol Chem, 2002
- By using Western blot we observed decreased levels of the  **AMPA**  receptor subunits GluR2 3 and GluR4 in  **trkB**  forebrains.  Ref: 12676531 Mol Cell Neurosci, 2003
